# Duck gasdermin E is a substrate of caspase-3/-7 and an executioner of pyroptosis

**DOI:** 10.3389/fimmu.2022.1078526

**Published:** 2023-01-10

**Authors:** Hanqing Li, Xin Wang, Lanjie Yu, Junwei Wang, Yongsheng Cao, Bo Ma, Wenlong Zhang

**Affiliations:** ^1^ College of Veterinary Medicine, Northeast Agricultural University, Harbin, Heilongjiang, China; ^2^ Northeastern Science Inspection Station, China Ministry of Agriculture Key Laboratory of Animal Pathogen Biology, Harbin, Heilongjiang, China

**Keywords:** duck, gasdermin E, caspaes-3, caspase-7, cleavage, pyroptosis

## Abstract

Gasdermin (GSDM)-mediated cell death is an ancient immune defensive mechanism that plays an essential role in bacteria, fungi, coral, teleost, and mammals. After being cleaved by proteases of hosts or pathogens, amino-terminal (NT) fragment of GSDMs (GSDM-NTs) form pores in the membrane structure of cells, thereby leading to pyroptotic cell death. However, the expression profile, activation mechanism and function of avian GSDMs have not been studied in depth yet. In the current study, genes encoding duck gasdermin E (duGSDME), caspase-3 (ducaspase-3) and ducaspase-7 were cloned from mRNA of a virus-challenged duck embryo. The cleavage of duGSDME by ducaspase-3/-7 was verified in the cell-free system and/or in human embryonic kidney cells (HEK293). Ducaspase-3/-7 could recognize and cleave duGSDME at 270DAVD273. Overexpression of duGSDME-NT (1-273aa) fragment led to pyroptosis-like morphological change, increased lactic dehydrogenase (LDH) release and propidium iodide uptake of HEK293 cells, which indicated that duGSDME-NTs could cause cell membrane damage. In addition, recombinantly expressed duGSDME-NT showed bactericidal activity to an enterotoxic *Escherichia coli* (F5+) strain. The expression level of duGSDME was low in duckling tissues. DHAV-3 challenge upregulated the expression of duGSDME and ducaspase-3 in different tissues and led to the activation of ducaspase-3 and cleavage of duGSDME. The results indicated that duGSDME is a substrate of ducapsase-3/-7, and duGSDME-NT can cause pyroptosis. In addition, duGSDME may play a role in the immune defense of ducks against infectious diseases after being cleaved by ducaspase-3. The current study provides essential information for further investigation of the mechanisms of avian innate immunity and avian diseases.

## Introduction

Members of the gasdermin (GSDM) protein family in mammals include Gasdermin A (GSDMA), Gasdermin B (GSDMB), Gasdermin C (GSDMC), Gasdermin D (GSDMD), Gasdermin E (GSDME) and DFNB59 (1). GSDM proteins, except for DFNB59, have been proven to be substrates of different proteases like caspases, granzymes, neutrophil elastase, and bacterial and viral proteases (2–10). The amino-terminal (N-terminal) fragment of the GSDM proteins produced by cleavage of GSDMs by some of these proteases can form pores in the plasma and mitochondrial membrane, thereby causing cell death (11, 12). The programmed cell death mediated by GSDM proteins has been named pyroptosis (1).

The activation mechanism and function of human and mouse GSDM proteins have been extensively studied. GSDME was originally named deafness, autosomal dominant 5 (DFNA5) because the gene encoding GSDME was found to be closely related to an autosomal dominant form of hearing impairment (1). Wang, et al. (5) showed that GSDME can be cleaved by caspase-3, thereby switching caspase-3-mediated apoptosis to pyroptosis.

In contrast to the well-studied human and mouse GSDME molecules, the GSDME molecules of other species have not been extensively studied. Jiang, et al. (13) found that coral GSDME can be cleaved by caspase-3, and the yielded GSDME N-terminal fragments can induce pyroptosis. Zebrafish GSDME can be cleaved by caspase-19b, caspase-3a/b, caspase-7 and caspase 8a/b and induce pyroptosis (14). *Cynoglossus semilaevis* (*C. semilaevis*) GSDME can be cleaved by caspase-1, caspase-3 and caspase-7 and induce pyroptosis (15). Recent studies showed that cadmium and molybdenum co-treatment significantly increased the mRNA level of GSDMA and GSDME in the brain, liver and kidney of ducks and duck renal tubular epithelial cells (16–20). However, the expression of duck GSDME (duGSDME) molecules in the tissues of ducks and the function of the molecule are largely unknown.

In the current study, genes encoding duGSDME, duck caspase-3 (ducaspase-3) and duck caspase-7 (ducaspase-7) were cloned from the mRNA of a duck embryo challenged with Newcastle disease virus (NDV). Cleavage of duGSDME by ducaspase-3 and ducaspase-7 and the cleavage site were confirmed in HEK293 cells and/or in the cell-free system. The pyroptosis-inducing ability of duGSDME cleavage products was determined in HEK293 cells. Finally, the expression and activation of duGSDME and ducaspase-3 in tissues of healthy ducklings and ducklings challenged with hepatitis A virus genotype 3 (DHAV-3) was determined using polyclonal antibodies against duGSDME and ducaspase-3. Our work provides information essential for understanding the process of programmed cell death in avian and explaining the pathogenesis of avian diseases.

## Materials and methods

### Ethics statement

The experimental protocol on animals was approved by the Ethics Committee on the Use and Care of Animals, Northeast Agricultural University, based on the Guide for the Care and Use of Laboratory Animals (Institute of Laboratory Animal Resources, Commission on Life Sciences, National Research Council, 2000). And all experiments were performed in accordance with the relevant guidelines and regulations.

### Genes and plasmids

The genes encoding duck GSDME (dugsdme), caspase-3 (ducaspase-3) and caspase-7 (ducaspase-7) were cloned from a duck embryo inoculated with Newcastle Disease Virus (NDV) (kindly provided by Prof. Bo Ma, Northeast Agricultural University). Briefly, RNA was extracted from the intestine tissue of the duck embryo. The cDNA was obtained by reverse transcription of mRNA using Oligo(dT). The duGSDME gene, ducaspase-3 gene and ducaspase-7 gene were amplified from the cDNA by PCR using the primers listed in **Table 1**.

**Table 1:** Primers used for genes cloning.

Genes	Primer sequence
Duck gsdme	5’-GGCTCTAAACGATTCTCC-3’
	5’-CTACGCAGTCCCACCTAA-3’
Duck caspase-3	5’-CCAGAATGACAGATGTAA-3’
	5’-TCTAGCAACGGAAGTA-3’
Duck caspase-7	5’-ATGTCAGGAGCTCACCAC-3’
	5’-ATAATCCAATCTCCACCC-3’

pET-30a(+), pcDNA3.1(+) and pECMV-3×flag-N expressing full-length or partial duGSDME, ducaspase-3 or ducaspase-7 were constructed using the primers listed in **Table 2** through the restriction sites indicated. The plasmids encoding mutants of duGSDME and ducaspase-3 were constructed using the primers listed in **Table 3** and Quick Mutation™ Site-Directed Mutagenesis Kit (Beyotime, China) according to the manufacturer’s instructions. All recombinant plasmids were verified by DNA sequencing. The characteristics of the plasmids were detailed in **Table 4**.

**Table 2:** Primers used for plasmid construction.

Plasmids	Primer sequence	Restriction site
pET-30a(+)-duGSDME	5’-CCGCATATGGCCGATAAGGTGC-3’	*Nde* I
	5’-CGGCTCGAGGCAACGGAAGTAAAATT-3’	*Xho* I
pET-30a(+)-ducaspase-3	5’-CGCCATATGACAGATGTAAAAG	*Nde* I
	5’-CGGCTCGAGATGTCCTGGGAAGAG-3’	*Xho* I
pET-30a(+)-ducaspase-7	5’-CGCCATATGTCAGGAGCTCACC-3’	*Nde* I
	5’-CGGCTCGAGGAAGTAAAGTTCCTTA-3’	*Xho* I
pET-30a(+)-duGSDME-NT	5’-CCGGAATTCATGTTTGCAAAAGCAACA-3’	*Eco*R I
	5’-CCGCTCGAGTTAATCAACAGCATCTGGCTG-3’	*Xho* I
pcDNA3.1(+)-ducaspase-3	5’-GCGATATCATGACAGATGTAAAAG 5’-CCGCTCGAGCTAGCAACGGAAGTAA	*Eco*R V *Xho* I
pcDNA3.1(+)-npducaspase-3	5’-GCGATATCATGTCAGGAATTCTGCCAGACT 5’-CCGCTCGAGCTAGCAACGGAAGTAA	*Eco*R V *Xho* I
pcDNA3.1(+)-ducaspase-7	5’-GCGATATCATGTCAGGAGCTCACC	*Eco*R V
	5’-CCGCTCGAGCTAGAAGTAAAGTTCC	*Xho* I
pECMV-3×FLAG-N-duGSDME	5’-CCCAAGCTTATGTTTGCAAAAGCAACA-3’	*Hin*d III
	5’-CCGCTCGAGCTACGCAGTCCCACCTAA-3’	*Xho* I
pcDNA3.1(+)-duGSDME-NT	5’-CCCAAGCTTATGTTTGCAAAAGCAACA-3’	*Hin*d III
	5’-CCGCTCGAGTTAATCAACAGCATCTGGCTG -3’	*Xho* I
pcDNA3.1(+)-duGSDMD-CT	5’-CCCAAGCTTATGAATGGGATGTACTCTG-3’	*Hin*d III
	5’-CCGCTCGAGTTAATCAACAGCATCTGGCTG-3’	*Xho* I

**Table 3:** Primers used for generating mutation in the eukaryotic expression plasmids.

Plasmids	Primer sequence
pET-30a(+)-duGSDME D270A	5’- GTTTCTGTATCAGCCAGCTGCTGTTGATAATGGGA-3’
	5’- TCCCATTATCAACAGCAGCTGGCTGATACAGAAAC-3’
pET-30a(+)-duGSDME D273A	5’-CAGCCAGATGCTGTTGCTAATGGGATGTACTCTGG-3’
	5’-CCAGAGTACATCCCATTAGCAACAGCATCTGGCTG-3’
pET-30a(+)-ducaspase-3 C170A	5’-CTTCTTCATTCAGGCTGCTAGAGGGACAGAACTA-3’
	5’TAGTTCTGTCCCTCTAGCAGCCTGAATGAAGAAG-3’

**Table 4:** Characteristics of the plasmids.

Plasmid names	Characteristics
pET-30a(+)-duGSDME	encodes a full length pGSDME, fused with His tag at the carboxyl terminal
pET-30a(+)-ducaspase-3	encodes a full length ducaspase-3, fused with His tag at the carboxyl terminal
pET-30a(+)-ducaspase-7	encodes a full length ducaspase-7, fused with His tag at the carboxyl terminal
pET-30a(+)-duGSDME-NT	encodes the 1-273 aa fragment of duGSDME (duGSDME-NT), fused with His tag at the amino terminal
pcDNA3.1(+)-ducaspase-3	encodes a full length ducaspase-3
pcDNA3.1(+)-ducaspase-7	encodes a full length ducaspase-7
pECMV-3×FLAG-N-duGSDME	encodes a full length duGSDME, fused with 3×FLAG tag at the amino terminal
pcDNA3.1(+)-duGSDME-NT	encodes the 1-273 aa fragment of duGSDME (duGSDME-NT)
pcDNA3.1(+)-duGSDMD-CT	encodes the 274-499 aa fragment of duGSDME (duGSDME-CT) (pGSDME-NT)
pET-30a(+)-duGSDME D270A	encodes a full length duGSDME, the Asp270 of the duGSDME is replaced with Ala, fused with His tag at the carboxyl terminal
pET-30a(+)-duGSDME D273A	encodes a full length duGSDME, the Asp273 of the duGSDME is replaced with Ala, fused with His tag at the carboxyl terminal
pET-30a(+)-ducaspase-3 C170A	encodes a full length ducaspase-3, the Cys273 of the duGSDME is replaced with Ala, fused with His tag at the carboxyl terminal

### Preparation of recombinant proteins

pET-30a(+)-dugsdme-His, pET-30a(+)-dugsdme D270A-His, pET-30a(+)-dugsdme D273A-His, pET-30a(+)-His-dugsdme-nt, pET-30a(+)-ducaspase3-His, pET-30a(+)-ducaspase3 C170A-His, and pET-30a(+)-ducaspase7-His were transformed into *Escherichia coli* (*E. coli*) Rosetta™ (DE3) competent cells (Novagen, USA). Isopropyl β-D-1-thioglycoside (IPTG) was used to induce the expression of the recombinant proteins. The expression of recombinant duGSDME-His (rduGSDME-His), recombinant duGSDME D270A-His (rduGSDME D270A-His), recombinant duGSDME D273A-His (rduGSDME D273A-His), recombinant His-duGSDME-NT (His-rduGSDME-NT), recombinant ducaspase-3-His (rducaspase-3-His), recombinant ducaspase-3 C170A-His (rducaspase-3 C170A-His), and recombinant ducaspase-7-His (rducaspase-7-His), was determined by sodium dodecyl sulfate-polyacrylamide gel electrophoresis (SDS-PAGE) and western blot assay.

The recombinant proteins were purified by the nickel ion affinity chromatography method. The purified proteins were dialyzed against PBS with 5% glycerol at 4 °C to remove the urea and/or imidazole. The protein concentration was determined by the BCA method. All the proteins were stored at −70 °C until use.

### Preparation and characterization of rabbit anti-duGSDME-NT and rabbit anti-ducaspase-3 antibodies

Two six-week-old female New Zealand white rabbits were used to prepare anti-duGSDME-NT antibody and rabbit anti-ducaspase-3 antibody. First, the two rabbits were immunized subcutaneously with His-rduGSDME-NT and rducaspase-3 C170A-His, respectively, four times. Each of the first three immunizations was performed at two-week intervals. A boosting immunization was carried out a week after the third immunization. The antigen dose for each immunization was 500 μg. Freund’s complete and incomplete adjuvants (Sigma-Aldrich) were used in the first immunization and last three immunizations, respectively. One week after the fourth immunization, the rabbits were sacrificed, and the blood was collected. The sera containing anti-His-rduGSDME-NT polyclonal antibodies or anti-rducaspase-3 C170A-His polyclonal antibodies were separated and stored at −70 °C. The two polyclonal antibodies were designated as anti-duGSDME-NT antibody and anti-ducaspase-3 antibody, respectively, in the current work.

The reactivity of the two antibodies to overexpressed duGSDME and ducaspase-3 was determined by western blot assays. Briefly, pECMV-3×flag-N-duGSDME and pcDNA3.1(+)-ducaspase-3 were transfected into human embryonic kidney cells (HEK293) using Linear PEI MAX 40K (Qifa Experimental Reagent Co., Ltd, China) according to the manufacturer’s instruction. After transfection, the cells were incubated at 37°C and harvested at indicated times. The cells were lysed in RIPA buffer (Beyotime, China) on ice for 15 min. After centrifugation at 10,000×g for 10 min, the supernatant of the lysates was collected. Proteins in the supernatant were separated by SDS-PAGE, followed by western blot assay using anti-duGSDME-NT antibody or anti-ducaspase3 antibody as primary antibody.

### Determination of the enzymatic activity of recombinantly expressed ducaspases-3/7

Ac-DEVD-pNA (Beyotime, China), a colorimetric substrate for caspase-3, was used to determine the enzymatic activity of recombinantly expressed ducaspases-3/7.

The prokaryotically expressed rducaspase-3-His and rducaspase-7-His were prepared and purified as described above.

Eukaryotic expression of ducaspase-3 was performed in HEK293 cells. Cells were transfected with vectors expressing ducaspase-3 or ducaspase-3 subunit. Cells were washed with ice-cold PBS (pH 7.2) and lysed on ice for 30 min with buffer containing 0.5% Triton X-100 and 2 mM EDTA. The soluble fraction was collected after centrifugation at 10,000 × g and 4°C for 15 min and used to determine the enzymatic activity of ducaspase-3.

The soluble fraction of the transfected HEK293 cells or the purified rducaspase-3-His and rducaspase-7-His were incubated with Ac-DEVD-pNA (final concentration 200 μM) in caspase reaction buffer (0.05 M HEPES, pH 7.5, 3 mM EDTA, 0.15 M NaCl, 0.005% [v/v] Tween-20 and 0.01M DTT) at 37°C. The absorbance of the mixture was measured at 405 nm wavelength.

### Cleavage of duGSDME by ducaspase-3/-7 in a cell-free system

The rduGSDME-His or rduGSDME-His mutants were mixed with different amounts of rducaspase-3-His or reducaspase-7-His. The cleavage reaction was carried out at 37°C for different periods.

To determine the relationship between rduGSDME cleavage and the enzymatic activity of rducaspases, in some experiments, Z-DEVD-FMK, a caspase-3 inhibitor (APExBIO, USA), was added to the reaction mixture to the indicated concentrations.

The products were separated by SDS-PAGE followed by Coomassie Brilliant Blue R250 staining or western blot analysis.

### Cleavage of duGSDME by ducaspase-3/-7 in HEK293 cells

HEK293 cells (1×10^6^ cells/well) were seeded in 6-well plates and cultured overnight at 37°C. Then the cells were transfected with pECMV-3×flag-N-duGSDME (2 μg/well) and vectors expressing npducaspase-3 (ducaspase-3 without prodomain) or ducaspase-7 (2 μg/well) and cultured at 37°C for indicated periods. The proteins of cells were analyzed by western blot assay using the anti-Flag antibody, anti-duGSDME-NT antibody or anti-ducaspase-3 antibody as primary antibodies.

### LDH assay

To determine the LDH release from cells caused by duGSDME fragments, HEK293 cells or Madin-Darby canine kidney (MDCK) cells (1×10^5^ cells/well) were seeded in 24-well plates and transfected with vectors expressing duGSDME, duGSDME-NT or duGSDME-CT. After transfection, cells were incubated at 37°C for different periods. The culture medium was collected. LDH release from the cells was measured using the LDH Cytotoxicity Assay Kit (Beyotime, China) according to the manufacturer’s instructions. The LDH release was calculated using the formula: percentage of LDH release =100 × (experimental sample − culture medium background)/(maximum LDH release − culture medium background).

### Microscope observation

An inverted microscope was used to observe the cells and capture static bright-field images.

### Propidium iodide staining assay

Transfected cells were fixed with 4% paraformaldehyde at 4°C for 20 min. Cells were then washed with ice-cold PBS and incubated with propidium iodide (PI) (1 μg/mL) (Beyotime, China) for 20 min at room temperature. An inverted fluorescence microscope was used to capture images.

### Western blot assay

The protein concentration of the samples was quantified using a BCA protein assay kit (Beyotime, China). The proteins were separated by SDS-PAGE and transferred to nitrocellulose (NC) membranes. The membranes were blocked with 5% skim milk plus 0.05% (v/v) Tween 20 (PBST) for 1 hour at room temperature. The membranes were incubated with the indicated primary antibodies overnight at 4°C and then incubated with horseradish peroxidase (HRP)-conjugated goat anti-mouse IgG (H+L) or HRP-conjugated goat anti-rabbit IgG for 1 h at room temperature. The membranes were washed three times with PBST for 5 min after each incubation step. Detection was performed using a chemiluminescent substrate (Beyotime, China) according to the manufacturer’s instructions.

### Bactericidal assay

Since His-rduGSDME-NT was expressed as inclusion bodies in *E. coli* Rossetta ™ (DE3) cells, it did not show bactericidal activity to the competent cells during the overexpression period. Therefore the current study investigated the bactericidal activity of the purified His-rduGSDME-NT. In brief, overnight culture of *Enterotoxic Escherichia coli* (ETEC) (F5+) (kindly provided by Prof. Dongfang Shi, Northeast Agricultural University) was washed three times with sterile PBS and then diluted to a final concentration of 200 colony forming unit (CFU)/mL. 200 μL diluted ETEC (F5+) was incubated with different concentrations of His-rduGSDME-NT at 37°C for 30 min and then spread on LB agar plate. The *E. coli* colonies on the plate were counted 24 hours later. As a control, pyolysin (PLO), a pore-forming toxin of *Trueperella pyogenes*, was recombinantly expressed (rPLO), purified (21) and incubated with ETEC (F5+).

### Determination of endogenous expression and activation of GSDME and caspase-3 in tissues of ducklings

Tissues of healthy and DHAV-3 challenged ducklings were kindly provided by Prof. Ma Bo. Proteins extracted from the tissues were separated by SDS-PAGE and analyzed by western blot assay with anti-duGSDME-NT antibody (1:3000) and anti-ducaspase-3 antibody (1:3000).

To confirm whether the protein bands on the blots indeed represent duGSDME molecules, in some experiments, rduGSDME-His was used to neutralize the duGSDME-specific antibody. Briefly, 10 μL of anti-duGSDME-NT antibody was diluted 1:100 in PBS and mixed with 200 μg rduGSDME-His, 200 μg rDHAV-3 VP1 (the recombinantly expressed DHAV-3 VP1 protein) or 200 μg rPLO, respectively. The mixture was incubated for 1 h at 37°C and then centrifuged at 10,000 × g and 4°C for 10 min. The supernatant was diluted to 30 mL with PBST and used as the primary antibody for western blot assay.

### Statistical analysis

One-way ANOVA test was enrolled in the current study as indicated in the figure legends. ****, *p*<0.0001, **, *p*<0.01, *, *p*<0.05, ns, not significant.

## Results

### GSDME, caspase-3 and caspase-7 are conserved among duck, goose and chicken

**Figure 1:**
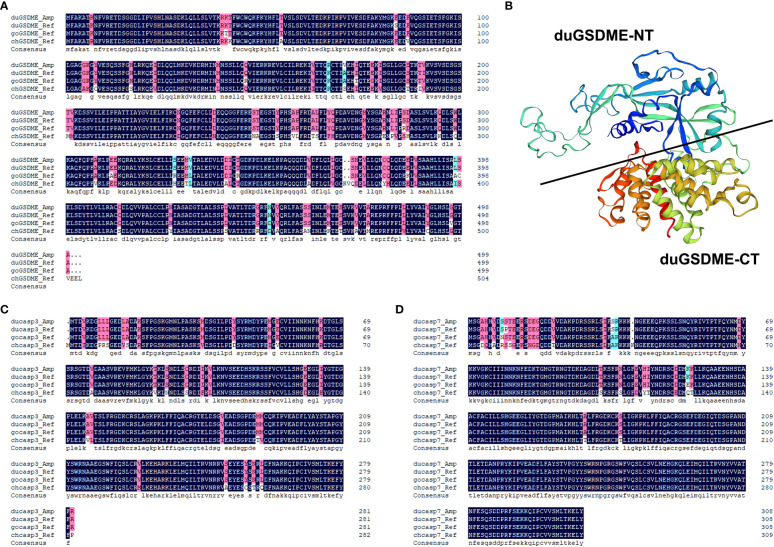
Analysis of the primary structures of duGSDME, ducaspase-3 and ducaspase-7. **(A)** Comparison of the primary structure of duGSDME deduced from the amplified dugsdme gene (duGSDME_Amp) with the primary structure of predicted avian GSDME proteins (duGSDME_Ref infers duck GSDME, goGSDME_Ref infers goose GSDME, chGSDME_Ref infers chicken GSDME). **(B)** The 3-D model of duGSDME molecule predicted by SWISS-model. The duGSDME molecule shows typical structure characteristics of gasdermin proteins, which have two domains (NT and CT) linked by an unfolded peptide. **(C)** Comparison of the primary structure of ducaspase-3 deduced from the amplified ducaspase-3 gene (ducasp3_Amp) with the primary structure of predicted avian caspase-3 proteins (ducasp3_Ref infers duck caspase-3, gocasp3_Ref infers goose caspase-3, chcasp3_Ref infers chicken caspase-3). **(D)** Comparison of the primary structure of ducaspase-7 deduced from the amplified ducaspase-7 gene (ducasp7_Amp) with the primary structure of predicted avian caspase-7 proteins (ducasp7_Ref infers duck caspase-7, gocasp7_Ref infers goose caspase-7, chcasp7_Ref infers chicken caspase-7).

The duGSDME, ducaspase-3 and ducaspase-7 genes were cloned and sequenced. duGSDME, ducaspase-3 and ducaspase-7 genes have open reading frames of 1500 bp, 849 bp and 930 bp, respectively. The primary structure of the protein encoded by the duGSDME gene shows 99.2%, 96.18% and 86.97% similarity to the predicted duGSDME protein (XP_005020592.3), goose GSDME (goGSDME) protein (XP_013027320.1) and chicken GSDME (chGSDME) protein (NP_001006361.2), respectively (**Figure 1A**). The structural model of duGSDME predicted by SWISS-MODEL showed that the duGSDME molecule had two structural domains (**Figure 1B**). The primary structure of the protein encoded by the ducaspase-3 gene shows 100%, 100% and 89.01% similarity to the predicted ducaspase-3 protein (XP_027312089.1), gocaspase-3 protein (XP_047934317.1) and chcaspase-3 protein (NP_990056.1), respectively (**Figure 1C**). The primary structure of the protein encoded by the ducaspase-7 gene shows 99.68%, 98.71% and 91.91% similarity to the predicted ducaspase-7 protein (XP_027315665.1), gocaspase-7 protein (XP_047916469.1), and chcaspase-7 protein (XP_040558934.1), respectively (**Figure 1D**). The results indicate that the genes cloned in the current study are indeed duGSDME, ducaspase-3 and ducaspase-7 genes, and the primary structures of GSDME, caspase-3 and caspase-7 are conserved among duck, goose and chicken.

### Prokaryotically expressed ducaspase-3 and ducaspase-7 show caspase-like enzymatic activity

**Figure 2:**
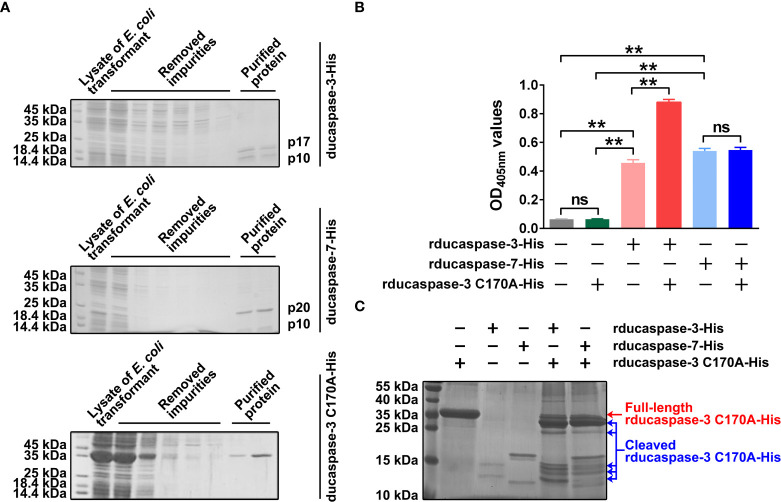
Preparation and characterization of prokaryotically expressed ducaspase-3 and ducapase-7 and ducaspase-3 C170A mutant. **(A)** The SDS-PAGE gels showed the purified rducaspase-3-His, rducaspase-7-His and rducaspase-3 C170A-His. Rducaspase-3-His and rducaspase-7-His were activated during the expression process, because the gels showed large subunits and small subunits of the two caspases, but not the full-length procaspases. Rducaspase-3 C170A-His was expressed in full-length form. Since the C170 is essential for the enzymatic activity of caspase-3, the C170A mutation completely blocked the self-activation of rducaspase-3. **(B)** Purified rducaspase-3-His, rducaspase-7-His and rducaspase-3 C170A-His were incubated with Ac-DEVD-pNA, respectively. Rducaspase-3-His and rducaspase-7-His, but not rducaspase-3 C170A-His, caused a significant increase in OD405nm values of the reaction mixtures compared with mock control, which indicated the caspase-like enzymatic activity of rducaspase-3-His and rducaspase-7-His. Incubation with rducaspase-3 C170A-His significantly increased the caspase-like enzymatic activity of rducaspase-3-His but not that of rducaspase-7-His. The experiment was repeated thrice. The data obtained in these experiments were statistically analyzed by one-way ANOVA test and shown as means ± SE (**, *p*=0.01, ns not significant). **(C)** The SDS-PAGE gel showed that both rducaspase-3-His and rducaspase-7-His could cleave rducaspase-3 C170A-His.

Ducaspase-3 and ducaspase-7 underwent self-activation when expressed in E. coli cells. When analyzed by SDS-PAGE, there were two polypeptides with approximate molecular weights of 17 kDa and 10 kDa in purified rducaspase-3-His and two polypeptides with approximate molecular weights of 19 kDa and 11 kDa in purified rducaspase-7-His. The molecular weights of these polypeptides were consistent with the molecular weights of the subunits of activated caspase-3 and caspase-7, respectively (**Figure 2A**). The rducaspase-3 C170A-His was expressed in full-length form (**Figure 2A**). When incubated with Ac-DEVD-pNA, purified rducaspase-3-His and rducaspase-7-His caused an increase in the OD405 nm value of the reaction mixture (**Figure 2B**). The results indicate that the prokaryotically expressed ducaspase-3 and ducaspase-7 exhibited caspase-like enzymatic activities.

Interestingly, when incubated with Ac-DEVD-pNA, the mixture of rducaspase-3-His and rducaspase-3 C170A-His was able to hydrolyze more Ac-DEVD-pNA than rducaspase-3-His, whereas the mixture of rducaspase-7-His and rducaspase-3 C170A-His mixture hydrolyzed a similar amount of Ac-DEVD-pNA as rducaspase-7-His (**Figure 2B**). SDS-PAGE showed that both rducaspase-3-His and rducaspase-7-His were able to cleave rducaspase-3 C170A-His (**Figure 2C**). The increased enzymatic activity of rducaspase-3-His after incubation with rducaspase-3 C170A-His may be due to the formation of new active rducaspase-3 in the mixture. The cleavage of rducaspase-3 C170A-His by rducaspase-3-His resulted in the release of the ducaspase-3 p10 subunit from rducaspase-3 C170A-His. The p10 subunits released from rducaspase-3-His C170A-His formed active rducaspase-3 with the free p20 subunits from rducaspase-3-His, which could then cleave Ac-DEVD-pNA. When incubated with rducaspase-7-His, the ducaspase-3 p10 subunit released from rducaspase-3 C170A-His could not form active rducaspase-3 because the ducaspase-3 p20 subunit was not present in the mixture.

### Anti-duGSDME-NT antibody and anti-ducaspase-3 antibody can recognize duGSDME and ducaspase-3 overexpressed in HEK293 cells

**Figure 3:**
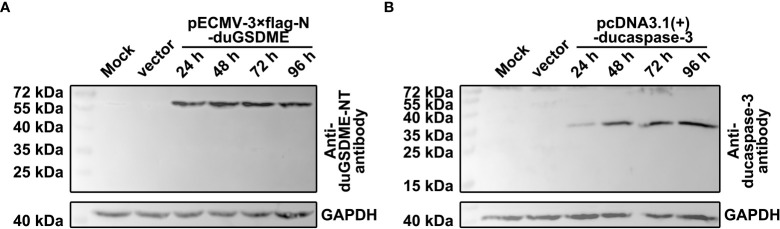
Determination of the reactivity of anti-duGSDME-NT antibody and anti-ducaspase-3 antibody to eukaryotically expressed duGSDME and ducaspase-3. Vectors expressing duGSDME and ducaspase-3 were transfected into HEK293 cells. The transfected cells were harvested 24, 48, 72 and 96 hours after the transfection. The reactivity of the antibodies to eukaryotically expressed duGSDME and ducaspase-3 was determined by western blot assays. The two antibodies could specifically recognize duGSDME **(A)** and ducaspase-3 **(B)**, respectively.

HEK293 cells were transfected with pECMV-3×flag-N-duGSDME or pcDNA3.1(+)-ducaspase-3. The blots showed that anti-duGSDME-NT antibody and anti-ducaspase-3 antibody specifically recognized the overexpressed duGSDME (**Figure 3A**) and ducaspase-3 (**Figure 3B**) in HEK293 cells, respectively.

### Ducaspase-3/-7 can cleave duGSDME in a cell-free system

**Figure 4:**
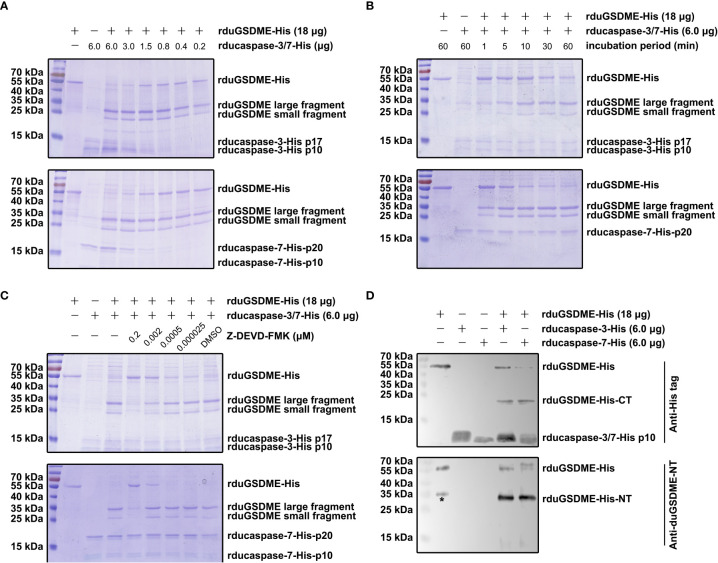
Determination of the cleavage of duGSDME by ducaspase-3 and ducaspase-7 in the cell-free system. **(A)** ducaspase-3 and ducaspase-7 cleaves duGSDME in a dose-dependent manner. Different amount of rducaspase-3-His or ducaspase-7-His was incubated with rduGSDME-His. Increasing the rducaspase-3-His or ducaspase-7-His in the reaction mixture increased the cleavage of rduGSDME-His. **(B)** Ducaspase-3 and ducaspase-7 cleaves duGSDME in a time-dependent manner. Rducaspase-3-His or rducaspase-7-His was incubated with rduGSDME-His, respectively, for different times. The cleavage of rduGSDME-His by rducaspase-3-His and rducaspase-7-His increased along with the extension of incubation time. **(C)** The cleavage of duGSDME depends on the caspase-like enzymatic activity of ducaspase-3 and ducaspase-7. Z-DEVD-FMK, a chemical inhibitor of caspase-3, attenuated the cleavage of rduGSDME-His by rducaspase-3-His and rducaspase-7-His in a dose-dependent manner. 0.2 μM Z-DEVD-FMK almost abolished the cleavage of rduGSDME-His by rducaspase-3-His and rducaspase-7-His completely. **(D)** Determination of the property of cleavage products of rduGSDME-His. The cleavage products of rduGSDME-His were separated by SDS-PAGE followed by western blot analysis. The 35 kDa and 25 kDa fragments reacted with the anti-duGSDME-NT antibody and the anti-His tag antibody, respectively (the * indicates nonspecific reaction between an unknown protein and the polyclonal antibody).

SDS-PAGE showed that rduGSDME-His can be cleaved by rducaspase-3-His and rducaspase-7-His (**Figure 4**). When incubated with rducaspase-3-His or rducaspase-7-His, rduGSDME-His was cleaved into two fragments with approximate molecular weights of 30 kDa (large fragment) and 25 kDa (small fragment). The cleavage of rduGSDME-His by rducaspase-3-His and rducaspase-7-His was rducaspases dose (**Figure 4A**) and incubation time (**Figure 4B**) dependent. Increasing the amount of rducaspases in the reaction mixture or extending the incubation time resulted in increased cleavage of rduGSDME-His.

The cleavage of rduGSDME-His by rducaspase-3-His and rducaspase-7-His depended on the enzymatic activity of the rducaspases, as Z-DEVD-FMK (inhibitor of caspase-3 and caspase-7) abolished rducaspases-mediated rduGSDME-His cleavage (**Figure 4C**).

Anti-duGSDME-NT antibody and anti-His tag antibody recognized the 30 kDa fragment (the larger fragment) and 25 kDa fragment (the small fragment), respectively (**Figure 4D**). The result indicates that the 30 kDa fragment is the amino-terminal fragment of rduGSDME-His, while the 25 kDa fragment is the carboxyl-terminal fragment.

### Ducaspase-3/-7 can cleave duGSDME in HEK293 cells

**Figure 5:**
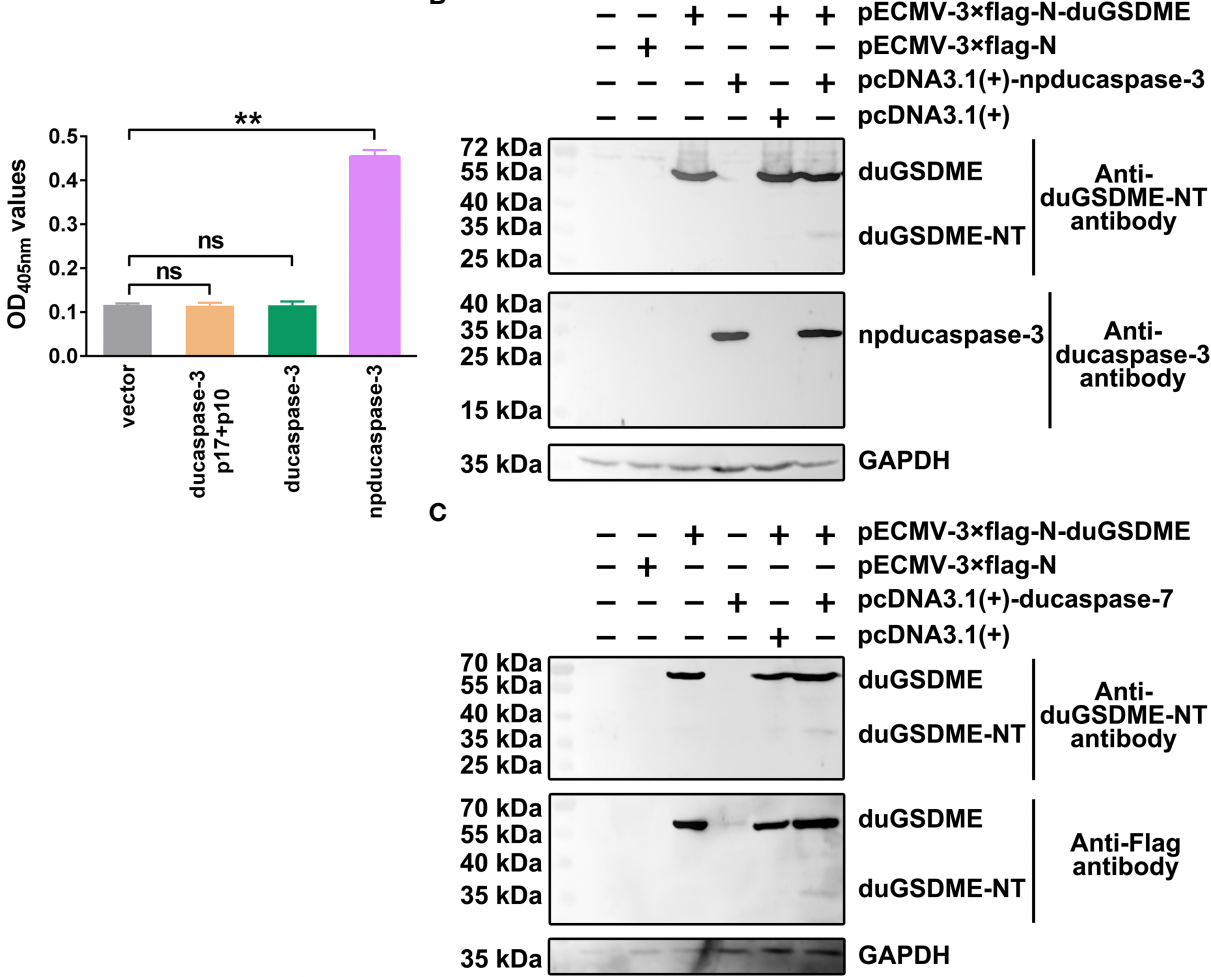
Determination of the cleavage of duGSDME by ducaspase-3 and ducaspase-7 in eukaryotic cells. **(A)** HEK293 cells were transfected with vectors expressing ducaspase-3, ducaspase-3 without prodomain (npducaspase-3), or ducaspase-3 p10 and ducaspase-3 p17. The whole cell lysates of the transfected cells were incubated with Ac-DEVD-pNA. Eukaryotically expressed npducaspase-3 showed caspase-like enzymatic activity, while ducaspase-3 did not. **, p=0.01; ns, not significant. **(B)** Cleavage of duGSDME by ducaspase-3 in HEK293 cells. Cells were co-transfected vectors expressing npducaspase-3 and duGSDME. Western blot assay showed a 35 kDa protein could be recognized by anti-duGSDME-NT antibody besides the 55 kDa full-length duGSDME in the co-transfection group, but not in other groups. **(C)** Cleavage of duGSDME by ducaspase-7 in HEK293 cells. Cells were co-transfected vectors expressing ducaspase-7 and duGSDME. Western blot assay showed a 35 kDa protein could be recognized by both anti-duGSDME-NT antibody and anti-Flag antibody besides the 55 kDa full-length duGSDME in the co-transfection group, but not in other groups.

To determine the enzymatic activity of ducaspase-3 overexpressed in HEK293 cells, the vectors expressing ducaspase-3 p17 subunit, ducaspase-3 p10 subunit, ducaspase-3 without primary domain (ducaspase-3 without primary domain, npducaspase-3) or full-length ducaspase-3 were transfected or co-transfected into HEK293 cells. The results showed that only npducaspase-3 could hydrolyze Ac-DEVD-pNA (**Figure 5A**).

In HEK293 cells, duGSDME could be cleaved by ducaspase-3 (**Figure 5B**) and ducaspase-7 (**Figure 5C**). In cells transfected with pECMV-3×flag-N-duGSDME and with pECMV-3×flag-N-duGSDME+pcDNA3.1(+), only the 55 kDa full-length duGSDME was detected by the anti-duGSDME-NT antibody and/or the anti-Flag antibody. In cells transfected with pECMV-3×flag-N-dugsdme +pcDNA3.1(+)-npducaspase-3 and with pECMV-3×flag-N-dugsdme +pcDNA3.1(+)-ducaspase-7, in addition to the 55 kDa duGSDME, another peptide with the approximate molecular weight of 30 kDa was also recognized by the anti-duGSDME-NT antibody or the anti-Flag tag antibody (**Figure 5B**). The results indicate that duGSDME can be cleaved by ducaspase-3 and ducaspase-7 in eukaryotic cells.

### Ducaspase-3/-7 cleaves duGSDME at the 270DAVD273 site

**Figure 6:**
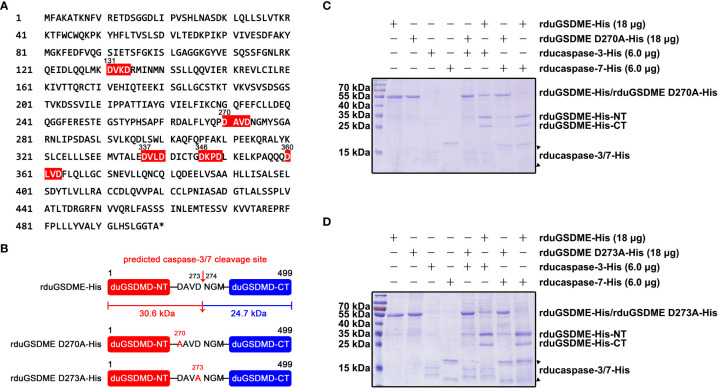
Determination of the cleavage site(s) recognized by ducaspase-3 and ducaspase-7 in duGSDME molecules. **(A)** Potential recognition and cleavage sites of caspase-3 and caspase-7 in duGSDME molecules. The motifs in duGSDME molecules with the composition of D-x-x-D (D infers aspartate, x infers any amino acid residue) are considered the potential cleavage sites recognized by ducaspase-3 and ducaspase-7 and marked by red. The * indicates the end of the primary structure of duGSDME. **(B)** Structure models of rduGSDME D270A-His and rduGSDME D273A-His mutants. In the two mutants, the D270 or D273 of the duGSDME molecule was replaced with an alanine, respectively. The two aspartates are the components of the predicted cleavage site of ducaspase-3 and ducaspase-7. The two cleavage products of duGSDME by ducaspase-3 and ducaspase-7 from the predicted site are 30.6 kDa and 24.7 kDa in their molecular weights, respectively. **(C)** The affection of the D270A mutation to the cleavage of duGSDME by ducaspase-3 and ducaspase-7. The D270A mutation completely abolished the cleavage of rduGSDME-His by rducaspase7-His and rducaspase-7-His according to the SDS-PAGE gel. **(D)** The affection of the D273A mutation to the cleavage of duGSDME by ducaspase-3 and ducaspase-7. The D273A mutation completely abolished the cleavage of rduGSDME-His by rducaspase7-His and rducaspase-7-His according to the SDS-PAGE gel.

Analysis of the primary structure of duGSDME molecules revealed five potential caspase-3/-7 cleavage sites, which were 131DVKD134, 270DAVD273, 337DVLD340, 346DKPD349, and 360DLVD363 (**Figure 6A**). Predictions based on these potential caspase-3/-7 cleavage sites yielded duGSDME-NT/-CT fragments with molecular weights of 15 kDa/40 kDa, 30 kDa/25 kDa, 37 kDa/18 kDa, 38 kDa/17 kDa, and 40 kDa/15 kDa, respectively. Given that the molecular weights of the products in the *in vitro* cleavage assays shown in **Figure 4** were approximately 30 kDa and 25 kDa, respectively, we speculated that 270 DAVD273 is the cleavage site of caspase-3/-7 in duGSDME molecules. To verify the speculation, we prepared rduGSDME D270A-His and rduGSDME D273A-His (**Figure 6B**). The results of the cleavage assays showed that the D270A and D273A mutations completely blocked the cleavage of duGSDME by ducaspase-3/-7 (**Figures 6C, D**). Therefore, the 270DAVD273 motif is the cleavage site of caspase-3/-7 in duGSDME molecules.

### DuGSDME-NT causes cell membrane damage and death in eukaryotic cells

**Figure 7:**
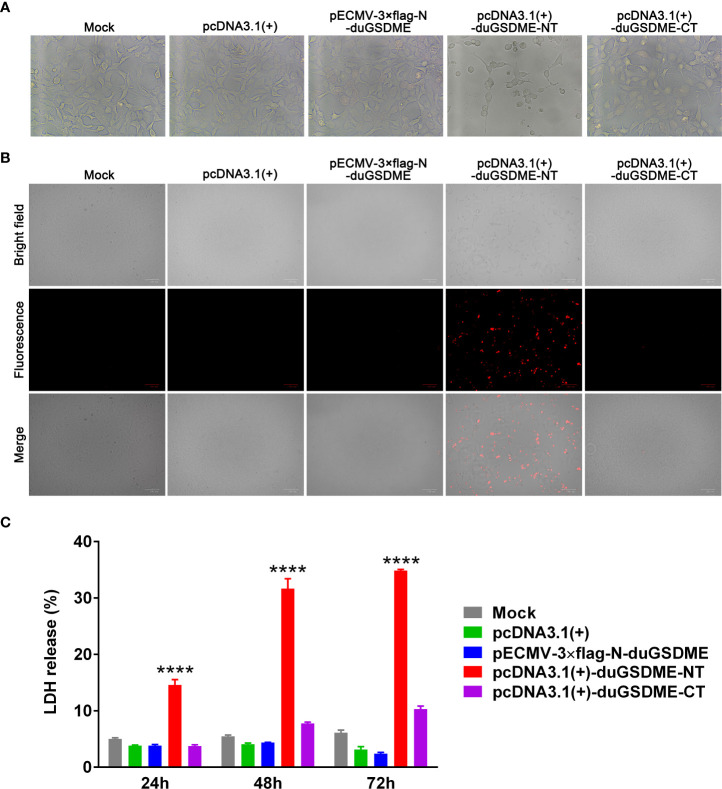
Determination of the ability of duGSDME-NT to cause cell membrane damage. **(A)** HEK293 cells were transfected with indicated plasmids. Transfected cells were observed under an inverted microscope. Overexpression of duGSDME-NT resulted in significant death and morphological change of the cells. The representative pictures that reflected the morphological features of the cells were taken. **(B)** Transfected HEK 293 cells were stained with PI (1 μg/mL) and observed under an inverted fluorescence microscope. Overexpression of duGSDME-NT, but not duGSDME and duGSDME-CT, resulted in increased uptake of PI by the cells. **(C)** LDH in culture supernatants of the transfected cells was determined 12, 24 and 48 hours after transfection. HEK293 cells expressing duGSDME-NT released more LDH than the cells expressing full-length duGSDME and duGSDME-CT. The experiment was repeated thrice. The data obtained in these experiments were statistically analyzed by one-way ANOVA test and shown as means ± SE (****, *p*=0.0001).

HEK293 cells were transfected with plasmids expressing duGSDME, duGSDME-NT or duGSDME-CT. Microscope observation showed that the cells expressing duGSDME-NT, but not the cells expressing duGSDME and duGSDME-CT, died significantly within 24 h after the transfection (**Figure 7A**).

The PI staining assay showed that the cells expressing duGSDME-NT were sensitive to PI staining. In contrast, almost no PI-positive cell was observed in cells expressing duGSDME and duGSDME-CT (**Figure 7B**).

The HEK293 cells expressing duGSDME-NT released significantly more LDH than the cells expressing duGSDME and duGSDME-CT (**Figure 7C**). A similar result was observed in MDCK cells although the expression of duGSDME-NT only caused the release of about 5% of total LDH (**Supplementary Figure 1A**) (the expression of duGSDME-NT caused the release of about 15% of total LDH in HEK293 cells within 24 hours as seen in **Figure 7C**). The western blot result in **Supplementary Figure 1B** confirmed the expression of duGSDME and duGSDME-NT in MDCK cells.

The above results suggest that duGSDME-NT can cause membrane permeabilization and cell death in HEK293 cells.

### DuGSDME-NT shows bactericidal effect against ETEC

**Figure 8:**
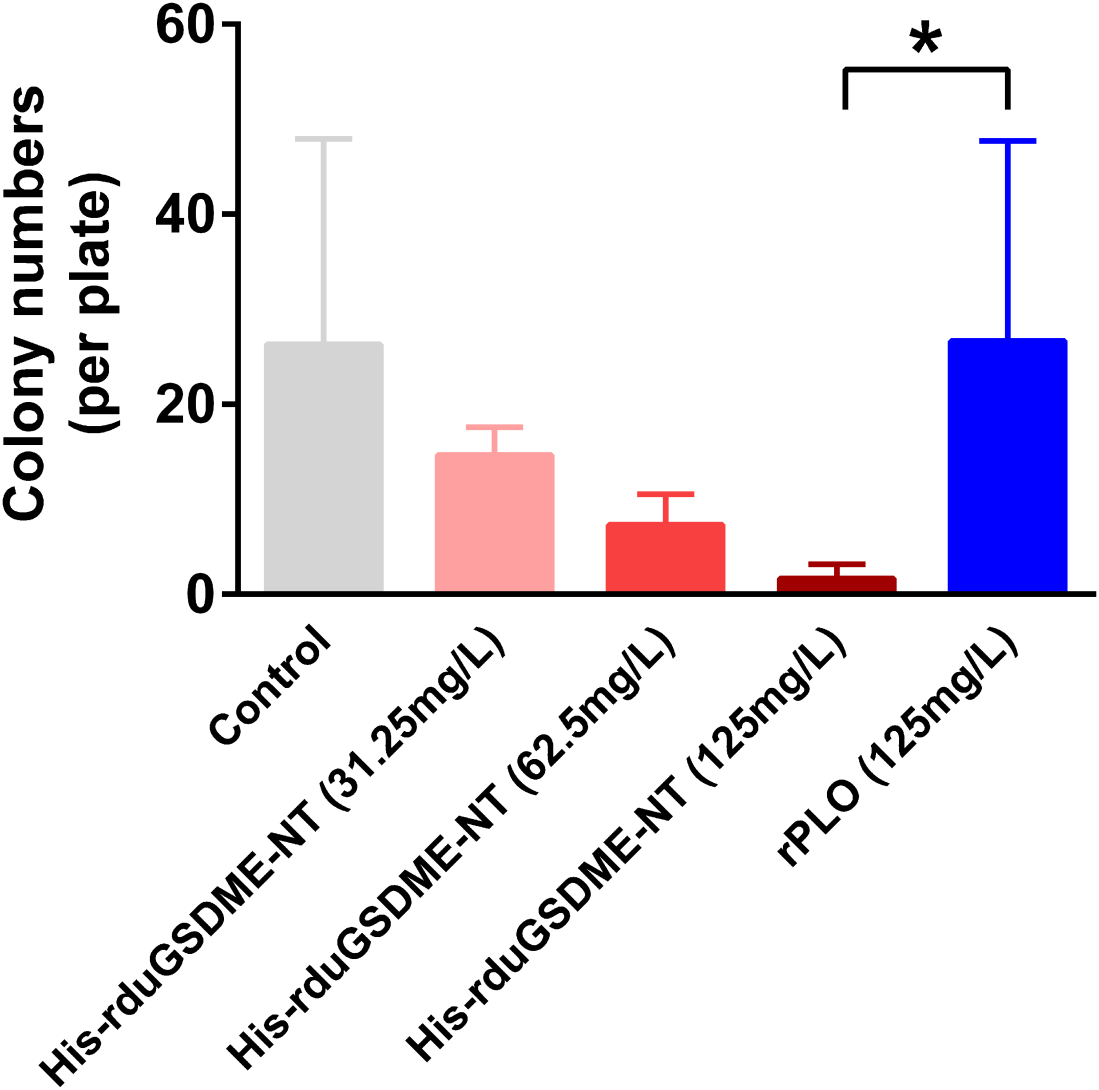
Determination of the bactericidal activity of duGSDME-NT. ETEC (F5+) was incubated with different amounts of purified His-rduGSDME-NT for 30 min and then spread on plates. The number of colonies was counted 24 hours later. ETEC (F5+) treated with purified rPLO served as a control. The colony number of the 125 mg/L His-rduGSDME-NT treated ETEC was significantly less than that of 125 mg/L rPLO treated ETEC. The experiment was repeated thrice. The data obtained in these experiments were statistically analyzed by one-way ANOVA test and shown as means ± SE (*, *p*=0.05).

When incubated with ETEC (F5+), purified His-rduGSDME-NT showed a bactericidal effect against this bacterium. The bactericidal effect of His-rduGSDME-NT on ETEC (F5+) was dose-dependent. Treatment with 125 μg/mL His-rduGSDME-NT significantly reduced the number of CFUs of ETEC (F5+) compared to 125 μg/mL rPLO treatment (**Figure 8**).

### DHAV-3 challenge activates ducaspase-3 and duGSDME in tissues of ducklings

**Figure 9:**
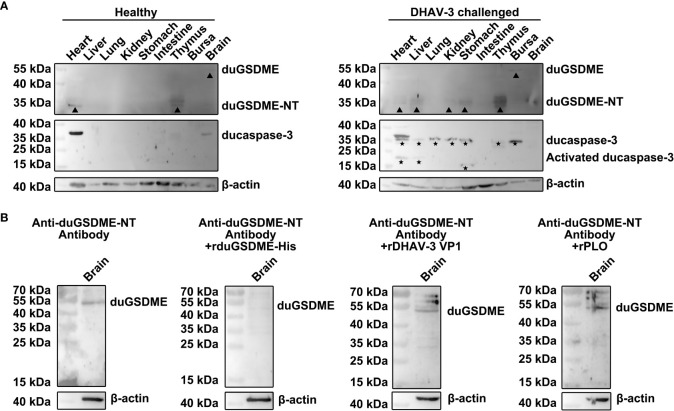
DHAV-3 challenge induces ducaspase-3 activation and duGSDME cleavage in ducklings. **(A)** duGSDME was expressed in the brain of the healthy duckling, although at an extremely low level. Expression of duGSDME-NT was detected in the heart and thymus of the healthy duckling. Ducaspase-3 was detected in the heart, brain and thymus of the healthy duckling, but no active ducaspase-3 was detected. DHAV-3 challenge resulted in the upregulation of full-length duGSDME in the bursa and duGSDME-NT in the liver, kidney, stomach and thymus of the duckling. DHAV-3 challenge also led to the upregulation of ducaspase-3 in the heart, liver, lung, kidney, stomach, thymus and bursa, and the activation of ducaspase-3 in the heart, liver and stomach of the duckling; **(B)** A 55 kDa protein in the brain of the healthy duckling was recognized by the anti-duGSDME-NT antibody. After the incubation with rduGSDME-His, the anti-duGSDME-NT serum failed to recognize the 55 kDa protein. In contrast, the incubation with rDHAV-3 VP1 and rPLO did not affect the recognition of the anti-duGSDME-NT serum to the 55 kDa protein.

Western blot assays showed that duGSDME and ducaspase-3 were expressed at low levels in the tissues of healthy ducklings (**Figure 9A**). Full-length duGSDME could be detected in the brains of ducklings.

DHAV-3 challenge significantly increased the levels of duGSDME-NT in ducklings’ liver, kidney, stomach, thymus and brain as well as the levels of full-length duGSDME in the bursa (**Figure 9A**). After DHAV-3 challenge, ducaspase-3 expression levels increased in the heart, liver, kidney, stomach, thymus, and bursa. Activation of ducaspase-3 in the heart, liver and stomach was observed in ducklings challenged with DHAV-3 (**Figure 9A**).

The specificity of the anti-duGSDME-NT antibody to recognize endogenous ducaspase was determined by Western blot assay. The 55 kDa protein in the brain of healthy ducklings was recognized by the anti-duGSDME-NT antibody. Incubation with rduGSDME-His completely abolished the ability of the anti-duGSDME-NT antibody to recognize this 55 kDa protein (**Figure 9B**). In contrast, rDHAV VP1 and rPLO did not block the recognition of the anti-duGSDME-NT antibody to the 55 kDa protein (**Figure 9B**). These results indicate that the 55 kDa protein recognized by the anti-duGSDME-NT antibody in the duck brain is indeed duGSDME. Therefore, the anti-duGSDME-NT antibody prepared in this study can specifically recognize endogenous duGSDME. Based on this result, we believe that the 55 kDa and 30 kDa bands in **Figure 9A** indeed represent the full-length duGSDME and duGSDME-NT, respectively.

## Discussion

GSDM proteins are executioners of pyroptosis in bacteria, fungi, corals, teleost and mammals (13, 22–24). However, avian GSDM proteins are not yet well studied. In the current study, the full-length genes encoding duGSDME, ducaspase-3, and ducaspase-7 were cloned from the mRNA of an NDV-infected duck embryo, but not from uninfected duck embryos (data not shown). A previous study showed that DHAV-3 infection resulted in the upregulation of mRNA levels of duGSDME, ducaspase-3, and ducaspase-7 in duck tissues (25). In addition to infection, molybdenum and cadmium treatment also induced upregulation of duGSDME and ducaspase-3 mRNA levels in duck tissues (20). The results of the present study are consistent with previous findings and suggest that the upregulation of duGSDME, ducaspase-3, and ducaspase-7 expression may be a general host response to different stimuli and play a role in host defense.

Unlike duck caspases, which are rarely studied, human and mouse caspases have been extensively studied (5, 26). Therefore, in the current study, we predicted the function and working mechanism of ducaspase-3 and ducaspase-7 based on data from human and mouse homologs. In humans and mice, D-x-x-D (x represents any amino acid residue) is the typical amino acid composition preferred by caspase-3 and caspase-7 (26, 27). After analysis, we found some tetrapeptides in duGSDME molecules whose composition matches the characteristic of typical caspase-3 and caspase-7 recognition sites. Therefore, in the following work, we focused on the ability of ducaspase-3 and ducaspase-7 to cleave duGSDME. However, whether other duck caspases can cleave duGSDME requires further investigation, as many other aspartic acid residues are in the duGSDME molecule, which may form potential caspase cleavage sites preferred by other ducaspases.

In the current study, we determined that duGSDME can be cleaved by ducaspase-3 and ducaspase-7 (**Figures 4**, **5**). Part of our results is consistent with a recent study which showed that duGSDME can be cleaved by ducaspase-3 (28). In addition, the current study showed that both ducaspase-3 and ducaspase-7 could recognize and cleave duGSDME at 270DAVD273 (**Figure 6**). The alignment result showed that the 270DAVD273 motif is conserved among chicken, duck and goose GSDME molecules (**Figure 1A**). Since caspase-3 and caspase-7 are also highly conserved among chicken, duck and goose (**Figures 1C, D**), we speculated that the cleavage of GSDME by caspase-3 and caspase-7 is a common event in poultry.

To determine the function of duGSDME cleavage products, duGSDME-NT (1-273aa of duGSDME) and duGSDME-CT (274-499aa of duGSDME) were overexpressed in HEK293 cells. Overexpression of duGSDME-NT in HEK293 cells caused cell membrane damage, as duGSDME-NT caused the significant release of LDH from the cells (**Figure 7**). In addition, HEK293 cells expressing duGSDME-NT, but not cells expressing duGSDME and duGSDME-CT, were positively stained by PI (**Figure 7**). Cheng and colleagues showed that in BHK-21 cells overexpressing both caspase-3 and duGSDME, duGSDME-NT was mainly located in the cytoplasm but not in the cell membrane (28). Since the formation of pores in the cell membrane by GSDM-NT fragments has been suggested to be a core event in pyroptotic cell death, the ability of duGSDME-NT to cause pyroptosis needs to be further investigated. These results also suggest that cell lines should be carefully selected when assessing the ability of GSDM-NTs to induce pyroptosis.

Overexpression of rduGDME-NT in competent *E. coli* cells did not kill bacterial cells because His-rduGSDME-NT molecules formed inclusion bodies rather than soluble proteins during IPTG-induced expression. However, purified rduGSDME-NT exhibited bactericidal activity when incubated with ETEC (F5+) to some extent (**Figure 8**). To exclude the possibility that the bactericidal activity of rduGSDME-NT was due to the high protein concentration, ETEC treated with recombinantly expressed PLO, a bacterial pore-forming toxin, was enrolled as a parallel control in this study. Compared to 125 mg/mL of rPLO, 125 mg/mL of rduGSDME-NT killed ETEC effectively. Previous studies have shown that human GSDMB-NT can kill *Shigella flexneri* (29), and GSDMD-NT can kill *E. coli* and *Staphylococcus aureus in vitro* (11). Teleost GSDME-NT showed bactericidal activity against competent *E. coli* cells when overexpressed under IPTG induction (15, 30). Our results provide evidence that GSDME can directly kill bacteria. The results suggest that when duGSDME is activated by ducaspase-3 or ducaspase-7, the derived duGSDME-NT may act as an executioner of intracellular bacterial killing in addition to its role in triggering pyroptosis. Unlike humans, ducks possess only GSDMA and GSDME genes. Therefore, the role of duGSDMs in the cell-autonomous immunity of the host may be more complex than that of any of the human GSDM.

Although studies have shown the presence of duGSDME mRNA in the tissues of adult ducks and ducklings (25, 28), the presence of duGSDME protein in tissues has not been determined. In the present study, using the anti-duGSDME-NT antibody, we confirmed that the expression level of full-length duGSDME in tissues of healthy ducklings was extremely low (**Figure 9A**). Cheng, et al. (28) showed that the duGSDME mRNA level was relatively higher in immune organs such as spleen, thymus, and bursa of healthy ducklings than in other organs such as heart, liver, and trachea. However, our results showed that full-length duGSDME was only detected in the brain of healthy ducklings and was undetectable in other tissues (**Figure 9A**). DHAV-3 challenge increased the expression of duGSDME-NT in the liver, kidney, stomach, thymus and brain of ducklings (**Figure 9A**). In addition, DHAV-3 challenge increased the expression and activation of ducaspase-3 in different tissues (**Figure 9A**). Ducaspase-3 activation and increased duGSDME-NT indicated that DHAV-3 challenge caused pyroptosis in the livers of ducklings. DHAV-3 challenge upregulated the expression of duGSDME and ducaspase-3 but did not activate ducaspase-3 in bursa (**Figure 9A**). A previous study showed that the viral load in the bursa is lower than that in the liver, heart, thymus, and kidney in ducklings challenged with DHAV-1/3 (31). The amount of DHAV-3 in the bursa may not be sufficient to activate ducaspase-3-mediated pyroptosis before the death of the ducklings. In the kidney, stomach, thymus, and brain of ducklings, while the expression level of duGSDME-NT increased upon DHAV-3 challenge, ducaspase-3 was not activated (**Figure 9A**). This is not surprising because, in human, granzyme B may also be an executioner of GSDME cleavage (32). Therefore, we speculate that there are other mechanisms to activate duGSDME-mediated pyroptosis in ducks. In addition, according to our results, ducaspase-7 is also an executioner of duGSDME cleavage (**Figure 4** and 5). However, we did not examine the expression and activation of ducaspase-7 in the tissues of ducklings because the current study did not prepare anti-ducaspase-7 antibodies. This issue should be investigated in further studies.

## Conclusion

In conclusion, this study determined the endogenous expression of duGSDME at the protein level, the cleavage of duGSDME by ducaspase-3 and ducaspase-7 at 270DAVD273, and the ability of duGSDME-NT to mediate pyroptosis and kill bacteria. Our results add to the existing knowledge about the mechanisms of avian innate immunity and provide new ideas for further studies on the pathogenic mechanisms of avian diseases.

## Data availability statement

The original contributions presented in the study are included in the article/**Supplementary Material**. Further inquiries can be directed to the corresponding authors.

## Ethics statement

The animal study was reviewed and approved by Ethics Committee on the Use and Care of Animals, Northeast Agricultural University.

## Author contributions

WZ designed the experiments, analyzed the results, supervised the work, and drafted the manuscript. HL, XW and LY performed the experiments. BM analyzed the results and supervised the work. YC and JW supervised the work. All authors contributed to the article and approved the submitted version.

## Supplementary material

Supplementary Figure 1 The overexpression of duGSDME-NT in MDCK cells leads to increase in LDH release. **(A)** LDH in culture supernatants of the transfected cells was determined 24 hours after transfection. MDCK cells expressing duGSDME-NT released more LDH than the cells expressing full-length duGSDME and duGSDME-CT. The experiment was repeated thrice. The data obtained in these experiments were statistically analyzed by one-way ANOVA test and shown as means ± SE (**, *p*=0.01, ****, *p*=0.0001, ns, not significant). **(B)** The expression of duGSDME and duGSDME-NT in transfected MDCK cells was determined by western blot assay. The triangles indicate the expressed flag-duGSDME; the arrow indicates the expressed duGSDME-NT; the bands marked by stars may represent the flag-duGSDME-NT derived from the expressed flag-duGSDME.
