# Supplementary figures and images for: Duck gasdermin E is a substrate of caspase-3/-7 and an executioner of pyroptosis
Source: Front Immunol. 2023 Jan 10;13:1078526. doi: 10.3389/fimmu.2022.1078526 (PMC9871645; doi:10.3389/fimmu.2022.1078526)

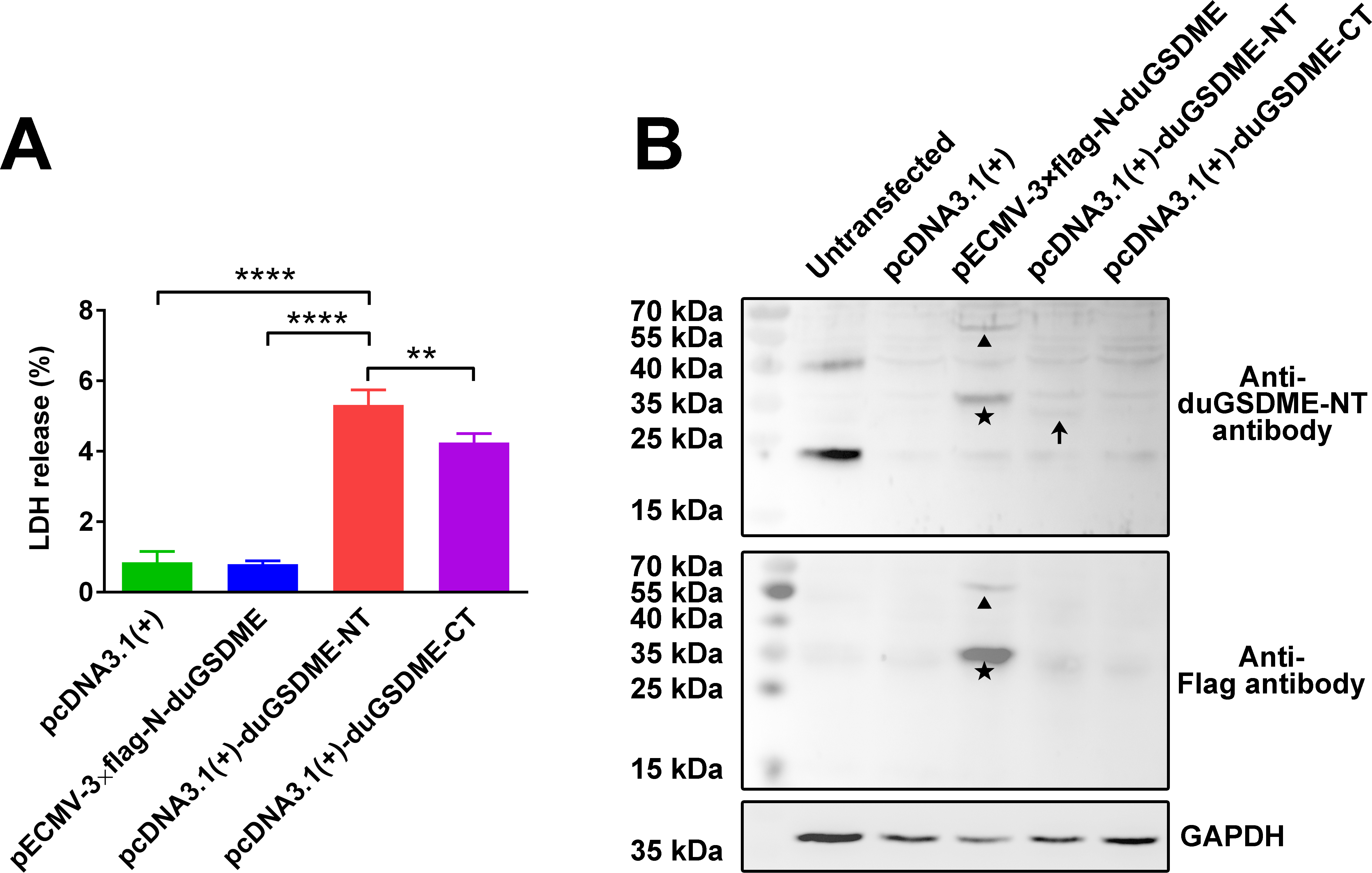

Supplement: Supplementary Figure 1 — The overexpression of duGSDME-NT in MDCK cells leads to increase in LDH release. (A) LDH in culture supernatants of the transfected cells was determined 24 hours after transfection. MDCK cells expressing duGSDME-NT released more LDH than the cells expressing full-length duGSDME and duGSDME-CT. The experiment was repeated thrice. The data obtained in these experiments were statistically analyzed by one-way ANOVA test and shown as means ± SE (**, p=0.01, ****, p=0.0001, ns, not significant). (B) The expression of duGSDME and duGSDME-NT in transfected MDCK cells was determined by western blot assay. The triangles indicate the expressed flag-duGSDME; the arrow indicates the expressed duGSDME-NT; the bands marked by stars may represent the flag-duGSDME-NT derived from the expressed flag-duGSDME. [file Image_1.tif]
